# Dupilumab Efficacy in Patients with Type 2 Asthma with and without Elevated Blood Neutrophils

**DOI:** 10.1155/2023/9943584

**Published:** 2023-10-19

**Authors:** Eugene R. Bleecker, Reynold A. Panettieri, Njira L. Lugogo, Jonathan Corren, Nadia Daizadeh, Juby A. Jacob-Nara, Yamo Deniz, Paul J. Rowe, Angela Khodzhayev, Xavier Soler, Thomas J. Ferro, Christopher N. Hansen

**Affiliations:** ^1^University of Arizona, College of Medicine, Division of Genomics and Precision Medicine, Department of Medicine, 1230 North Cherry Street, Suite 251, Tucson, AZ 85721, USA; ^2^Child Health Institute of New Jersey, Rutgers, The State University of New Jersey, New Brunswick, NJ, USA; ^3^University of Michigan, Ann Arbor, MI, USA; ^4^David Geffen School of Medicine at UCLA, Los Angeles, CA, USA; ^5^Sanofi, Cambridge, MA, USA; ^6^Sanofi, Bridgewater, NJ, USA; ^7^Regeneron Pharmaceuticals Inc., Tarrytown, NY, USA

## Abstract

**Introduction:**

Elevated neutrophil counts in blood, sputum, or lung have been associated with poor clinical outcomes and more severe disease in patients with type 2 asthma. In the phase 3 LIBERTY ASTHMA QUEST (NCT02414854), add-on dupilumab 200 and 300 mg every 2 weeks compared with matched placebo significantly reduced severe asthma exacerbations and improved forced expiratory volume in 1 s (FEV_1_) in patients with uncontrolled, moderate-to-severe asthma. This *post hoc* analysis explored the efficacy of dupilumab in patients with type 2 asthma enrolled in QUEST with or without elevated blood neutrophil counts.

**Methods:**

Annualized severe exacerbation rates during the 52-week treatment period and least-squares mean change from baseline in FEV_1_ over time were evaluated for patients with elevated type 2 biomarkers at baseline (blood eosinophils ≥ 150 cells/*µ*L or fractional exhaled nitric oxide (FeNO) ≥ 20 ppb; and eosinophils ≥ 300 cells/*µ*L or FeNO ≥ 50 ppb) and low (<4,000 cells/*µ*L) or high (≥4,000 cells/*µ*L) neutrophil counts.

**Results:**

Dupilumab significantly reduced annualized severe exacerbation rates compared with placebo during the 52-week treatment period in patients with elevated type 2 biomarkers, irrespective of baseline neutrophil count (*P* < 0.0001 for all comparisons). Significant improvements in FEV_1_ versus placebo were observed as early as Week 2 and over the 52-week treatment period, irrespective of baseline neutrophil count (*P* < 0.001 for all comparisons). Safety findings were similar across all subgroups, regardless of neutrophil counts at baseline.

**Conclusions:**

Dupilumab treatment significantly reduced annualized severe exacerbation rates and improved lung function in patients with uncontrolled, moderate-to-severe, type 2 asthma, irrespective of baseline blood neutrophil count. This trial is registered with NCT02414854.

## 1. Introduction

Asthma is a heterogeneous and chronic inflammatory disease characterized by a spectrum of overlapping phenotypes [1–3]. Patients vary in their clinical and inflammatory presentations across these different asthma profiles. The type 2 inflammatory asthma phenotype, which is estimated to affect at least 50% of all asthma patients [4], is characterized by elevated type 2 biomarkers, including blood and sputum eosinophils, immunoglobulin E (IgE), and fractional concentration of exhaled nitric oxide (FeNO) [5–7]. However, patients often present with overlapping non-type 2 biomarkers, such as elevated neutrophils [8, 9].

Neutrophils may play a key role in asthma, attracting other immune cells and contributing to mucus hypersecretion and increased smooth muscle responsiveness [10–12]. Elevated neutrophils can occur in patients with or without type 2 asthma [2, 8] and have been associated with poor outcomes across a number of asthma phenotypes [13–16]. Neutrophil counts in blood, sputum, or lungs have been associated with disease pathogenesis and are predictive of both disease severity and patient outcomes in patients with severe asthma [15]. The combination of high levels of sputum or blood neutrophils and eosinophils has also been associated with reduced pulmonary function and increased risk of hospitalization in patients with moderate-to-severe asthma [15–20]. Subsequently, these patients often have a heavy disease burden and may remain unresponsive to treatment.

It has been suggested that non-type 2 mechanisms, including neutrophilic inflammation, may directly affect patient outcomes and the efficacy of asthma treatment [8, 21–23]. Because asthma phenotypes and associated inflammatory profiles overlap [8], it is important to establish therapeutic efficacy across subgroups. As an example, responses to anti-inflammatory therapy, including inhaled corticosteroids (ICS), may be reduced in patients with noneosinophilic asthma or neutrophilic inflammation [6, 24–26]. However, mechanistic data suggest that interleukin (IL)-4 and IL-13 can inhibit neutrophil effector functions, aiding in the transition from a proinflammatory role to an anti-inflammatory role [27, 28], and monoclonal antibodies targeting IL-4 and/or IL-13 may, therefore, not only inhibit type 2 mechanisms but also may interfere with neutrophilic inflammation, thus improving treatment efficacy.

Dupilumab, a fully human monoclonal antibody, blocks the shared receptor component for IL-4 and IL-13, key and central drivers of type 2-mediated inflammation [29, 30] in multiple diseases. In the phase 3 LIBERTY ASTHMA QUEST study (QUEST; NCT02414854), add-on dupilumab 200 or 300 mg every 2 weeks (q2w), compared with matched placebo, significantly reduced severe asthma exacerbations and improved prebronchodilator forced expiratory volume in 1 s (FEV_1_), and was well tolerated in the overall population of patients with uncontrolled, moderate-to-severe asthma [31]. Greater treatment effects were observed in patients with elevated type 2 biomarkers at baseline (blood eosinophils ≥ 150 cells/*μ*L or FeNO ≥ 25 parts per billion (ppb)) [31]. This *post hoc* analysis of QUEST aims to assess the consistency of dupilumab treatment within the heterogeneous type 2 asthma populations to more precisely evaluate the potential impact of non-type-2 biomarkers such as neutrophils on the efficacy of dupilumab in patients with type 2 asthma with and without elevated blood neutrophil counts at baseline.

## 2. Methods

### 2.1. Study Design and Patients

QUEST was a phase 3, multicenter, randomized, double-blind, placebo-controlled, parallel-group trial evaluating the safety and efficacy of dupilumab in patients aged ≥12 years with uncontrolled, moderate-to-severe asthma. Dupilumab is approved in the USA as an add-on maintenance treatment in patients with moderate-to-severe asthma aged ≥6 years with an eosinophilic phenotype or with oral corticosteroid-dependent asthma, and in Europe to treat patients with uncontrolled, severe asthma aged ≥6 years [32, 33]. Between May 2015 and September 2016, eligible patients were randomized 2:2:1:1 to receive add-on 200 or 300 mg subcutaneous dupilumab or matched placebo q2w for a total of 52 weeks. Eligible patients were aged ≥12 years and met key inclusion criteria of current treatment with medium-to-high dose ICS plus ≤ 2 additional controllers, prebronchodilator FEV_1_ ≤ 80% (≤90% if aged 12–17 years) predicted normal value, FEV_1_ reversibility of 12% and 200 mL, a score of ≥1.5 on 5-point Asthma Control Questionnaire (ACQ-5), and worsening asthma in the past year. Patients were eligible for enrollment irrespective of a minimum baseline blood eosinophil count or levels of type 2 inflammatory biomarkers. Key exclusion criteria included weight <30 kg, comorbid lung diseases, severe asthma exacerbation, and current smoking, smoking cessation <6 months prior to the study, or history of >10 pack-years. Full details of the study design and inclusion and exclusion criteria have been published previously [31]. The study was conducted in accordance with the Declaration of Helsinki and the International Conference on Harmonisation Good Clinical Practice guideline, with applicable local regulations. The protocol and informed consent/assent forms were approved by institutional review boards and ethics committees, as appropriate, before the start of the study. All patients (or parents/legal guardians for adolescents) provided written informed consent, and assent was obtained from adolescent patients in line with local standard practice.

Only patients with type 2 asthma (defined as having baseline blood eosinophils ≥ 150 cells/*µ*L and/or FeNO ≥ 20 ppb per Global Initiative for Asthma (GINA) guidelines [5]) were included in the current analysis.

### 2.2. Endpoints

**Figure 1:**
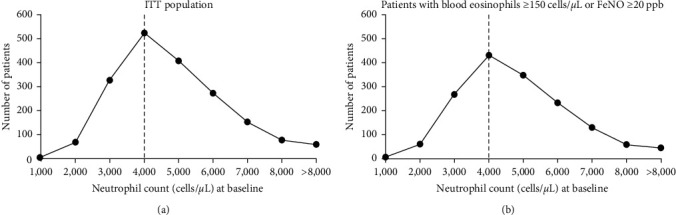
Distribution of baseline neutrophil counts in patients in the ITT population and in those with blood eosinophils ≥ 150 cells/*µ*L or FeNO ≥ 20 ppb at baseline. Category width = 1,000 cells/*µ*L. FeNO = fractional exhaled nitric oxide; ITT = intention-to-treat; ppb = parts per billion.

Efficacy endpoints assessed in this analysis were annualized severe exacerbation rates over the 52-week treatment period and change from baseline in prebronchodilator FEV_1_ over time. For analysis, patients were stratified into subgroups based on baseline levels of type 2 biomarkers and neutrophils: eosinophils ≥ 150 cells/*µ*L or FeNO ≥ 20 ppb (type 2-150/20) AND low or high neutrophil count or eosinophils ≥ 300 cells/*µ*L or FeNO ≥ 50 ppb (type 2-300/50) AND low or high neutrophil count. It should be noted here that the cutoff of eosinophils ≥ 150 cells/*µ*L includes all patients with blood eosinophils ≥ 150 cells/*µ*L (and not only those with 150–300 cells/*µ*L), and FeNO ≥ 20 ppb refers to all patients with FeNO of 20 ppb or greater, and not only those with FeNO 20–50 ppb; thus, patients in type 2-300/50 subgroups were by definition also included in the less strict type 2-150/20 subgroup. The threshold for high and low neutrophil count was set at ≥4,000 and <4,000 cells/*µ*L, respectively, based on previous studies and the recent Severe Asthma Research Program (SARP) analyses [17]. To further assess appropriateness of the 4,000 cells/*µ*L threshold in the current study population, median (95% confidence interval (CI)) blood neutrophil counts were analyzed and the number of patients in different neutrophil count categories at baseline (categorized in blocks of 1,000, such as 1,000 to <2,000; 2,000 to <3,000, etc.) was assessed in the intention-to-treat (ITT) population and in patients with eosinophils ≥ 150 cells/*µ*L or FeNO ≥ 20 ppb (Figure 1).

Safety was measured in terms of treatment-emergent adverse events (TEAEs) and serious AEs (SAEs) throughout the study. Safety data were evaluated by treatment (dupilumab or placebo) for patients with or without clinically defined treatment-emergent neutropenia (<1,500 cells/*µ*L).

### 2.3. Statistical Analysis

For this analysis, data were pooled within treatment types (combined dupilumab 200 and 300 mg q2w and combined matched placebo groups). Annualized severe exacerbation rates over the 52-week treatment period were assessed using a negative binomial model, with the total number of events with onset from randomization up to visit 18 or the last contact date (whichever came earlier) as the response variable. The pooled treatment groups, age, region (pooled country), baseline eosinophil strata, baseline ICS dose level, and the number of severe exacerbation events within 1 year prior to the study were included in the model as covariates and the log-transformed standardized observation duration as an offset variable. Change from baseline in FEV_1_ was assessed using a linear mixed-effects model with repeated measures, with change from baseline in prebronchodilator FEV_1_ up to Week 52 as the response variable, and treatment, age, sex, baseline height, region (pooled country), baseline eosinophil strata, baseline ICS dose level, visit, treatment-by-visit interaction, baseline prebronchodilator FEV_1_ value, and baseline-by-visit interaction as covariates. Additional covariates—the subgroup, subgroup-by-treatment interactions, and subgroup-by-treatment-by-visit interaction—were added for the interaction *P*-value model. A *P* value of <0.05 was considered statistically significant for the comparisons between dupilumab and placebo. The number of type 2 patients by neutrophil count categories at baseline and the safety results were summarized descriptively. In addition, restricted cubic spline regression models with a maximum of 4 knots (*k*) regressions were implemented to explore the relationship between the outcomes (annualized severe exacerbation rates and change from baseline in prebronchodilator FEV_1_ at Week 52) and baseline biomarker (blood eosinophils and FeNO) levels. The spline models for the annualized severe exacerbation outcome used a penalized negative binomial model, with the pooled treatment groups, age, region (pooled country), baseline ICS dose level (medium or high), number of severe exacerbation events within 1 year prior to the study, baseline biomarker (eosinophils or FeNO), and baseline biomarker-by-treatment (eosinophils or FeNO) interaction as covariates, and log-transformed standardized observation duration as an offset variable. Similarly, the spline models for the change from baseline in prebronchodilator FEV_1_ at Week 52 outcome used a penalized regression with the pooled treatment groups, age, sex, baseline height, region (pooled country), baseline ICS dose (medium or high), and baseline prebronchodilator FEV_1_, baseline biomarker (eosinophils or FeNO), and baseline biomarker-by-treatment (eosinophils or FeNO) interaction as covariates. The negative binomial model and mixed-effects model with repeated measures were analyzed using SAS v9.4, and spline regression analyses were performed using R-3.6.2.

## 3. Results

### 3.1. Baseline Characteristics

In total, 1,582 patients were included in the current analysis. Patients' mean age was similar across the analysis subgroups; however, a higher proportion of adolescents were included in the subgroups with low neutrophil counts (<4,000 cells/*µ*L) (Table 1). Baseline pre- and postbronchodilator FEV_1_ were similar across the groups (1.70–1.87 and 2.06–2.31 L, respectively), and the mean number of severe exacerbations in the previous year ranged from 1.91 to 2.32. Approximately half of the patients in each of the analyzed subgroups were taking high-dose ICS at baseline. Baseline blood eosinophil and FeNO levels were broadly similar across treatment groups, regardless of neutrophil count at baseline.

Overall, median (95% CI) blood neutrophil counts in the QUEST ITT population were 4,050 cells/*µ*L (3,940–4,210) and 4,060 cells/*µ*L (3,950–4,180) in the placebo and dupilumab treatment arms, respectively, with similar blood neutrophil counts in the type 2-150/20 (placebo: 4,080 (3,950–4,260) cells/*µ*L; dupilumab: 4,050 (3,930–4,170) cells/*µ*L) and type 2-300/50 (placebo: 3,950 (3,700–4,100) cells/*µ*L; dupilumab: 4,115 (3,920–4,280) cells/*µ*L) groups, further confirming the appropriateness of the chosen cutoff of 4,000 neutrophils/*µ*L.

### 3.2. Annualized Severe Exacerbation Rates

**Figure 2:**
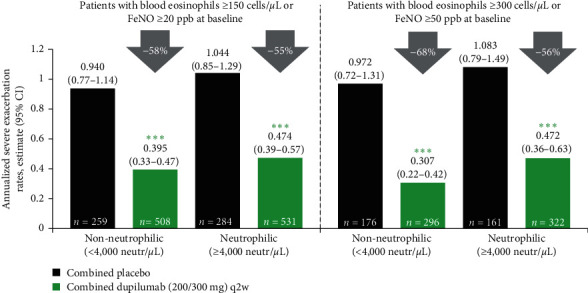
Annualized severe exacerbation rates over the treatment period in patients with elevated type 2 biomarkers with or without elevated neutrophil counts at baseline. CI = confidence interval; FeNO = fractional exhaled nitric oxide; neutr = neutrophils; ppb = parts per billion; q2w = every 2 weeks.  ^*∗∗∗*^*P* < 0.001.

Over the 52-week treatment period, significantly lower (*P* < 0.001) annualized severe exacerbation rates were observed in patients treated with dupilumab versus placebo across the type 2-150/20 and type 2-300/50 subgroups, irrespective of neutrophil count (Figure 2). In patients with type 2-150/20 asthma at baseline, 58% (low neutrophil group) and 55% (high neutrophil group) reductions in annualized severe exacerbation rates were seen for dupilumab versus placebo. Similar findings were observed in patients with type 2-300/50 at baseline (68% (low neutrophil count) and 56% (high neutrophil count) lower rates for dupilumab vs. placebo). Across both the type 2-150/20 and type 2-300/50 subgroups, no significant difference in reductions was observed regardless of neutrophil count at baseline (type 2-150/20: *P*_int_ = 0.6056; type 2-300/50: *P*_int_ = 0.1787).

### 3.3. Change from Baseline in Prebronchodilator FEV_1_

**Figure 3:**
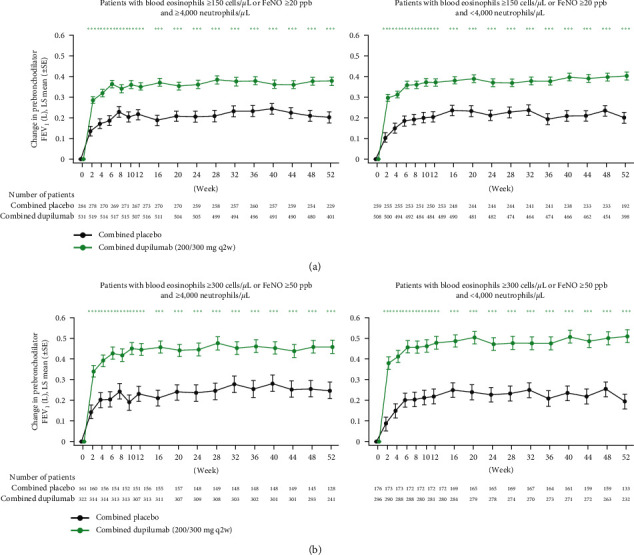
Change from baseline in prebronchodilator FEV_1_ over the treatment period in patients with elevated type 2 biomarkers with or without elevated neutrophil counts at baseline. FeNO = fractional exhaled nitric oxide; FEV_1_ = forced expiratory volume in 1 s; LS = least squares; ppb = parts per billion; q2w = every 2 weeks; SE = standard error.  ^*∗∗∗*^*P* < 0.001.

Significantly greater improvements (*P* < 0.001) in the least squares (LS) mean change from baseline in prebronchodilator FEV_1_ were observed for dupilumab versus placebo at all assessed timepoints, irrespective of baseline eosinophil, FeNO, or neutrophil level (Figure 3). By Week 2, LS mean difference versus placebo was 0.19–0.15 L in patients with type 2-150/20 asthma at baseline and 0.29–0.20 L in patients with type 2-300/50 at baseline with low and high neutrophil counts, respectively. By the end of the study at Week 52, LS mean differences versus placebo were 0.20 and 0.18 L in patients with type 2-150/20 asthma at baseline and 0.31 and 0.21 L in patients with type 2-300/50 asthma at baseline with low and high neutrophil counts, respectively. These changes were not significantly different between low and high neutrophil groups (type 2-150/20: *P*_int_ = 0.5797; type 2-300/50: *P*_int_ = 0.0899).

### 3.4. Regression Analysis of Severe Exacerbations and Change from Baseline in Prebronchodilator FEV_1_ at Week 52 against Baseline Eosinophil or Baseline FeNO Levels

**Figure 4:**
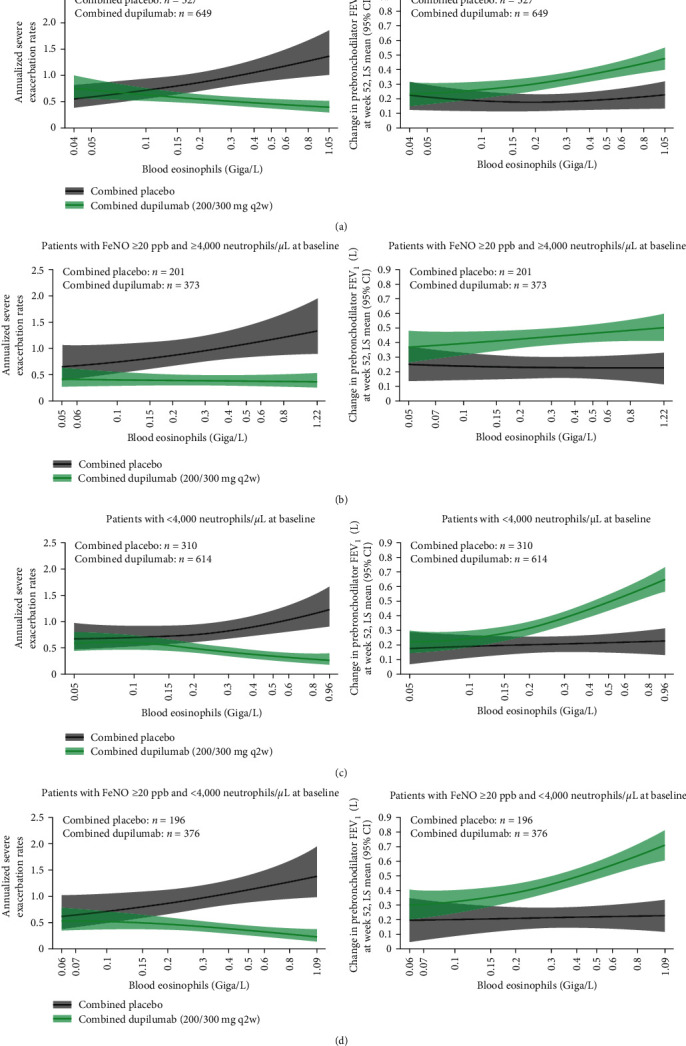
(a–d) Change in annualized severe exacerbation rates over the treatment period and change from baseline in prebronchodilator FEV_1_ at Week 52 by baseline blood eosinophils (Giga/L) in patients with ≥4,000 neutrophils/*µ*L at baseline, with FeNO ≥ 20 ppb AND ≥4,000 neutrophils/*µ*L at baseline, with <4,000 neutrophils/*µ*L at baseline, and with FeNO ≥ 20 ppb AND <4,000 neutrophils/*µ*L at baseline. CI = confidence interval; FeNO = fractional exhaled nitric oxide; FEV_1_ = forced expiratory volume in 1 s; LS = least squares; ppb = parts per billion; q2w = every 2 weeks.

Regression analysis indicated that dupilumab reduced severe exacerbations and improved prebronchodilator FEV_1_ in patients with ≥4,000 neutrophils/*µ*L and in patients with ≥4,000 neutrophils/*µ*L and FeNO ≥ 20 ppb at baseline, with greater benefits seen in those patients with higher blood eosinophil counts at baseline (Figure 4). Similar results were observed in patients with <4,000 neutrophils/*µ*L at baseline (Figure 4). Greater benefits were also seen in patients with higher FeNO levels at baseline, regardless of the neutrophil count at baseline (Figures S1 and S2, available in this article's Online Repository). Furthermore, patients with blood eosinophils ≥ 300 cells/*µ*L or FeNO ≥ 50 ppb showed a similar pattern, although benefits may be more variable, as suggested by a wider CI.

### 3.5. Safety

In the primary analysis of QUEST, the overall rates of TEAEs were similar in the combined placebo (83.1%) and dupilumab (81.0%) groups (SAEs, 8.4% and 8.2%, respectively). The most frequent AE occurring in ≥5% of patients and at higher rates among patients who received dupilumab was injection-site reaction (16.8% of patients in combined dupilumab vs. 7.9% in combined placebo groups). Viral upper respiratory tract infections (URTI) were the next most frequent AE in all groups [31]. Pneumonia was the most frequent SAE, observed in four patients (0.3%) in the combined dupilumab arm and two patients (0.3%) in the combined placebo arm. AEs leading to death total were observed in five patients (0.4%) who received dupilumab (one at the lower dose and four at the higher dose) and three patients (0.5%) who received placebo; all were considered by the investigator to be unrelated to the intervention.

In patients with and without neutropenia, as analyzed here, overall rates of TEAEs and SAEs also were similar between dupilumab and placebo (Tables S1 and S2, available in this article's Online Repository). The most frequently reported TEAE by Preferred Term in all groups was viral URTI. In the combined dupilumab versus combined placebo groups, viral URTI was reported in 21.8% versus 26.0%, respectively, in patients with neutrophils <1,500 cells/*µ*L and in 17.7% versus 18.7%, respectively, in patients with neutrophils ≥1,500 cells/*µ*L. In patients with neutrophils <1,500 cells/*µ*/L, any-class SAE was reported in four patients (2.7%) in the combined dupilumab group and one patient (1.3%) in the combined placebo group, and in patients with neutrophils ≥1,500 cells/*µ*L, in 36 patients (3.2%) in the combined dupilumab group and 23 (4.1%) in the combined placebo group. The most frequent SAE was pneumonia, which occurred in one patient (0.7%) with neutropenia and three patients (0.3%) without neutropenia in the combined dupilumab group and two patients (0.4%) in the combined placebo group (both patients without neutropenia).

## 4. Discussion

In this *post hoc* analysis of the phase 3 QUEST study, treatment with dupilumab, which blocks the shared receptor for IL-4 and IL-13 signaling, significantly reduced annualized severe exacerbation rates and improved lung function in patients with uncontrolled, moderate-to-severe, GINA-defined type 2 asthma [5], irrespective of blood neutrophil count at baseline. Comparable results were seen in patients with blood eosinophil counts ≥ 300 cells/*µ*L or FeNO ≥ 50 ppb at baseline with and without elevated neutrophil counts. The findings of this analysis indicate that the efficacy of dupilumab is consistent across patients with type 2 asthma who also present with neutrophil counts ≥4,000 or <4,000 cells/*µ*L at baseline, and they suggest that reducing IL-4/IL-13 signaling may reduce patients' clinical disease burden regardless of neutrophil count.

To our knowledge, this is the first analysis that describes the efficacy of a biologic that targets underlying type 2 inflammation by baseline neutrophil count in patients with asthma. Within the spectrum of asthma, elevated neutrophils can occur with or without type 2 inflammation. In a cluster analysis of patients in the SARP program, type 2 asthma patients with severe asthma who had the poorest lung function, frequent hospitalization, and uncontrolled symptoms despite high-dose oral corticosteroid use also had elevated blood neutrophils [10]. In a separate analysis assessing the relationship between type 2 eosinophilic and non-type 2 mechanisms in patients with asthma, higher IL-6 levels tended to be positively correlated with blood neutrophil count and are thought to be a driver of non-type 2 mechanisms that can coincide with elevation of eosinophils, IgE, or nitric oxide production [8, 34]. Asthma is a complex disease comprising a spectrum of phenotypes independent of type 2 biomarkers. In patients with asthma, elevations of non-type-2 signals such as IL-6 correspond with higher blood neutrophil counts, obesity, a lower percent predicted FEV_1_, increased asthma-related hospitalizations, and increased use of systemic corticosteroids. These trends were also observed in nonobese patients, indicating that IL-6 may be associated with more severe symptoms, irrespective of body mass index [8, 23]. Given this association with increased non-type 2 pathways such as IL-6, future analyses should consider its overlap within type 2 asthma patients and immune pathway contributions because assessment of levels of type 2 biomarkers and non-type 2 biomarkers may aid in identifying overlapping asthma endotypes and potential responsiveness to treatment in patients with persistent severe asthma [8, 35]. It has become clear that a deeper understanding of the heterogeneity of severe asthma is crucial, and an unmet need lies in the application of subgroup profiling to the responses to biologics. Future analyses are needed across asthma biologics to determine predictive and prognostic capacity based on various patient clusters. In the present study, we suspect that monoclonal antibodies targeting IL-4 and/or IL-13 may inhibit central processes that improve outcomes in patients with or without mixed phenotypes that may have a reduced capacity for response to baseline asthma therapies. The results of the current analysis indicate that dupilumab treatment improves lung function and reduces the rate of severe exacerbations in patients irrespective of neutrophil count (using a cutoff of 4,000 cells/*µ*L, in line with previous SARP analysis) [16, 36], although, since biomarkers were not evaluated postbaseline, we cannot elucidate whether there are any mechanistic differences between patients with and without elevated neutrophils. Future analysis of the potential prognostic utility of non-type 2 biomarkers in predicting treatment response should be considered to evaluate outcomes of a biologic targeting type 2 inflammation in patients with mixed or overlapping phenotypes.

One limitation of the current analysis is that it was performed *post hoc*, and the study was not specifically designed to assess the differences in efficacy across neutrophil counts. Therefore, sputum, which could provide more direct insight into neutrophilic airway inflammation and its potential interaction with airway eosinophils, was not collected. Smaller studies may be better tailored to evaluate sputum and other mechanistic outcomes. Our findings suggest that it would be of interest to evaluate induced sputum in smaller mechanistic studies because of its relation to the effects of biological treatment in this patient population. Since this was a large regulatory clinical trial, collection and analysis of induced sputum was not feasible, as in most clinical settings, blood biomarkers were more readily obtainable.

## 5. Conclusions

In summary, type 2 asthma comprises a spectrum of heterogeneity that includes patients with and without elevated blood neutrophils. Treatment with dupilumab resulted in consistent improvements in lung function in patients with uncontrolled, moderate-to-severe asthma across the type 2 spectrum, irrespective of mixed heterogeneity as described by blood neutrophil levels. Comparable responses were observed in patients with blood eosinophils ≥ 300 cells/*µ*L or FeNO ≥ 50 ppb at baseline and higher or lower blood neutrophil levels. As other new biomarkers are validated for non-type 2 asthma endotypes, future studies should evaluate predictive and prognostic outcomes after treatment with targeted type 2 biologics within overlapping asthma endotypes.

## Figures and Tables

**Table 1:** Baseline characteristics for patients with elevated type 2 biomarkers with or without elevated neutrophil counts at baseline.

	Patients with blood eosinophils ≥150 cells/*µ*L or FeNO ≥ 20 ppb at baseline				Patients with blood eosinophils ≥300 cells/*µ*L or FeNO ≥ 50 ppb at baseline			
	Neutrophils < 4,000 cells/*µ*L		Neutrophils ≥ 4,000 cells/*µ*L		Neutrophils < 4,000 cells/*µ*L		Neutrophils ≥ 4,000 cells/*µ*L	
	Combined PBO (*n* = 259)	Combined DPL (*n* = 508)	Combined PBO (*n* = 284)	Combined DPL (*n* = 531)	Combined PBO (*n* = 176)	Combined DPL (*n* = 296)	Combined PBO (*n* = 161)	Combined DPL (*n* = 322)
Age, mean (SD) (years)	48.1 (15.9)	46.7 (16.3)	47.7 (14.3)	48.1 (14.4)	47.9 (16.0)	46.3 (16.1)	46.6 (14.9)	47.0 (14.2)
Age 12–18 years, *n* (%)	23 (8.9)	37 (7.3)	10 (3.5)	16 (3.0)	16 (9.1)	24 (8.1)	7 (4.3)	11 (3.4)
Female sex, *n* (%)	165 (63.7)	302 (59.4)	179 (63.0)	323 (60.8)	111 (63.1)	173 (58.4)	96 (59.6)	201 (62.4)
Prebronchodilator FEV_1_, mean (SD) (L)	1.79 (0.62)	1.86 (0.65)	1.74 (0.56)	1.75 (0.58)	1.74 (0.63)	1.87 (0.65)	1.80 (0.59)	1.70 (0.56)
Percent predicted FEV_1_, mean (SD) (L)	59.4 (13.1)	60.0 (12.9)	57.2 (13.8)	57.0 (13.9)	58.1 (13.9)	60.2 (12.6)	57.9 (13.8)	55.4 (14.4)
Postbronchodilator FEV_1_, mean (SD) (L)	2.17 (0.73)	2.26 (0.77)	2.16 (0.69)	2.12 (0.70)	2.14 (0.73)	2.31 (0.77)	2.22 (0.71)	2.06 (0.67)
FEV_1_ reversibility, mean (SD) (%)	25.1 (17.4)	27.2 (23.4)	27.2 (19.0)	26.1 (20.6)	26.2 (18.0)	27.4 (23.5)	26.3 (20.0)	26.0 (19.5)
Severe asthma exacerbations^†^ in past year, mean (SD)	2.22 (1.98)	1.91 (1.61)	2.23 (1.84)	2.18 (2.86)	2.32 (2.08)	2.07 (1.79)	2.20 (1.67)	2.28 (2.21)
High-dose ICS at baseline, *n* (%)	134 (51.7)	229 (45.1)	152 (53.5)	297 (55.9)	87 (49.4)	130 (43.9)	89 (55.3)	179 (55.6)
With ongoing atopic medical condition,^‡^*n* (%)	215 (83.0)	427 (84.1)	241 (84.9)	438 (82.5)	145 (82.4)	258 (87.2)	141 (87.6)	260 (80.7)
ACQ-5 score, mean (SD)	2.75 (0.74)	2.66 (0.73)	2.75 (0.77)	2.88 (0.83)	2.80 (0.75)	2.62 (0.73)	2.80 (0.74)	2.90 (0.86)
AQLQ(S) global score, mean (SD)	4.33 (1.04)	4.42 (1.03)	4.20 (1.00)	4.20 (1.10)	4.26 (1.03)	4.45 (1.03)	4.16 (0.94)	4.20 (1.10)
Blood eosinophil count, median (IQR) (cells/*µ*L)	340.0 (200.0–540.0)	300.0 (180.0–495.0)	280.0 (190.0–525.0)	320.0 (180.0–560.0)	440.0 (340.0–685.0)	440.0 (340.0–650.0)	480.0 (370.0–780.0)	490.0 (340.0–710.0)
Total IgE, median (IQR) (IU/mL)	207.5 (75.0–456.0)	192.0 (69.0–522.0)	212.0 (73.0–503.5)	167.5 (77.0–499.0)	244.0 (82.0–507.0)	214.0 (94.0–574.0)	298.0 (102.5–636.0)	214.0 (88.0–587.0)
FeNO, median (IQR) (ppb)	32.0 (20.0–54.0)	29.0 (19.0–53.0)	29.00 (18.0–49.0)	27.00 (18.0–43.0)	42.0 (24.0–65.0)	43.5 (25.0–68.0)	40.0 (22.0–67.0)	33.0 (21.0–60.0)

*Note*: ACQ-5 = 5-item Asthma Control Questionnaire; AQLQ(S) = standardized Asthma Quality of Life Questionnaire; DPL = dupilumab; FeNO = fractional exhaled nitric oxide; FEV_1_ = forced expiratory volume in 1 s; ICS = inhaled corticosteroids; IgE = immunoglobulin E; IQR = interquartile range; PBO = placebo; ppb = parts per billion; SD = standard deviation. ^†^Severe asthma exacerbation prior to the study is defined as any treatment with one or more systemic (oral or parenteral) steroid bursts for worsening asthma, or hospitalization, or an emergency/urgent medical care visit for worsening asthma. ^‡^A patient is considered to have an ongoing atopic medical condition if they have any of the following ongoing conditions: atopic dermatitis, allergic conjunctivitis or rhinitis, eosinophilic esophagitis, food allergy, hives, or has baseline total IgE ≥ 100 IU/mL and at least one aero-antigen–specific IgE is positive (≥0.35 IU/mL) at baseline.

## Data Availability

Qualified researchers may request access to patient level data and related study documents, including the clinical study report, study protocol with any amendments, blank case report form, statistical analysis plan, and dataset specifications. Patient level data will be anonymized, and study documents will be redacted to protect the privacy of our trial participants. Further details on Sanofi's data sharing criteria, eligible studies, and process for requesting access can be found at: https://www.vivli.org.

## Abbreviations

AE: Adverse event
FeNO: Fractional exhaled nitric oxide
FEV_1_: Forced expiratory volume in 1 s
ICS: Inhaled corticosteroid
ITT: Intention-to-treat
IL-4/IL-13: Interleukin-4/interleukin-13
LS: Least squares
ppb: Parts per billion
q2w: Every 2 weeks
SAE: Serious adverse event
SARP: Severe Asthma Research Program.

## Additional Points

*Key Messages*. (i) Studies have demonstrated poor prognosis in patients with type 2 asthma and elevated neutrophil counts; (ii) treatment with dupilumab was effective in patients with uncontrolled, moderate-to-severe, type 2 asthma, irrespective of blood neutrophil counts; and (iii) patients with type 2 asthma and elevated blood neutrophils had consistently beneficial responses to dupilumab treatment.

## Supplementary Materials

Supplementary Materials Table S1: Treatment-emergent AEs and SAEs in patients with <1,500 neutrophils/*µ*L (neutropenia) or ≥1,500 neutrophils/*µ*L at baseline—safety population. Table S2: Treatment-emergent SAEs in patients with <4,000 or ≥4,000 neutrophils/*µ*L at baseline—safety population. Figure S1: Change in annualized severe exacerbation rates over the treatment period and change from baseline in prebronchodilator FEV_1_ at Week 52 by baseline FeNO (ppb) in patients with ≥4,000 neutrophils/*µ*L at baseline, and in patients with blood eosinophils ≥ 150 cells/*µ*L AND ≥4,000 neutrophils/*µ*L at baseline. Figure S2: Change in annualized severe exacerbation rates over the treatment period and change from baseline in prebronchodilator FEV_1_ at Week 52 by baseline FeNO (ppb) in patients with <4,000 neutrophils/*μ*L at baseline, and in patients with blood eosinophils ≥ 150 cells/*µ*L AND <4,000 neutrophils/*µ*L at baseline.
