# Supplementary material for: Dupilumab Efficacy in Patients with Type 2 Asthma with and without Elevated Blood Neutrophils
Source: J Immunol Res. 2023 Oct 19;2023:9943584. doi: 10.1155/2023/9943584 (PMC10602700; doi:10.1155/2023/9943584)

# **Online Repository Materials**

# **Dupilumab Efficacy in Patients With Type 2 Asthma With and Without Elevated Blood Neutrophils**

Eugene R. Bleecker, MD, Reynold A. Panettieri, Jr., MD, Njira L. Lugogo, MD,
Jonathan Corren, MD, Nadia Daizadeh, PhD, Juby A. Jacob-Nara, MD, Yamo Deniz, MD,

Paul J. Rowe, MD, Angela Khodzhayev, MD, Xavier Soler, MD,
Thomas J. Ferro, MD, Christopher N. Hansen, PhD, MBA

This appendix has been provided by the authors to give readers additional information about their work.

Contents

[**Online Repository Materials** 0](#_Toc121763242)

[**Dupilumab Efficacy in Patients With Type 2 Asthma With and Without Elevated Blood Neutrophils** 0](#_Toc121763243)

[**TABLE E1.** Treatment-emergent AEs and SAEs in patients with <1500 neutrophils/µL (neutropenia) or ≥1500 neutrophils/µL at baseline – safety population 2](#_Toc121763244)

[**TABLE E2.** Treatment-emergent AEs in patients with <4000 or ≥ 4000 neutrophils/uL at baseline – safety population 3](#_Toc121763245)

[**FIGURE E1.** Change in annualized severe exacerbation rates over the treatment period and change from baseline in pre-bronchodilator FEV_1_ at Week 52 by baseline FeNO (ppb) in patients with ≥4000 neutrophils/µL at baseline, and in patients with blood eosinophils ≥150 cells/µL AND ≥4000 neutrophils/µL at baseline. 9](#_Toc121763246)

[**FIGURE E2.** Change in annualized severe exacerbation rates over the treatment period and change from baseline in pre-bronchodilator FEV_1_ at Week 52 by baseline FeNO (ppb) in patients with <4000 neutrophils/μL at baseline, and in patients with blood eosinophils ≥150 cells/µL AND <4000 neutrophils/µL at baseline. 10](#_Toc121763247)

## **TABLE E1.** Treatment-emergent AEs and SAEs in patients with <1500 neutrophils/µL (neutropenia) or ≥1500 neutrophils/µL at baseline – safety population

| **Patients with at least one AE/SAE** | **Treatment-emergent AEs, n (%)** | | | | **Treatment-emergent SAEs, n (%)** | | | |
| --- | --- | --- | --- | --- | --- | --- | --- | --- |
|  | **Neutrophils <1500 cells/µL** | | **Neutrophils ≥1500 cells/µL** | | **Neutrophils <1500 cells/µL** | | **Neutrophils ≥1500 cells/µL** | |
|  | **Combined PBO**  **(n = 77)** | **Combined DPL**  **(n = 174)** | **Combined PBO**  **(n = 557)** | **Combined DPL**  **(n = 1116)** | **Combined PBO**  **(n = 77)** | **Combined DPL**  **(n = 174)** | **Combined PBO**  **(n = 557)** | **Combined DPL**  **(n = 1116)** |
| Any class | 57 (74.0) | 92 (62.6) | 388 (69.7) | 704 (63.1) | 1 (1.3) | 4 (2.7) | 23 (4.1) | 36 (3.2) |
| Viral upper respiratory tract infection | 20 (26.0) | 32 (21.8) | 104 (18.7) | 198 (17.7) | - | - | - | - |
| Upper respiratory tract infection | 8 (10.4) | 13 (8.8) | 78 (14.0) | 133 (11.9) | - | - | 1 (0.2) | 0 |
| Bronchitis | 14 (18.2) | 12 (8.2) | 75 (13.5) | 132 (11.8) | - | - | 0 | 2 (0.2) |
| Pneumonia | 0 | 2 (1.4) | 9 (1.6) | 12 (1.1) | 0 | 1 (0.7) | 2 (0.4) | 3 (0.3) |
| Atypical pneumonia | 1 (1.3) | 0 | 1 (0.2) | 0 | 1 (1.3) | 0 | - | - |
| Chronic sinusitis | 0 | 1 (0.7) | 1 (0.2) | 0 | 0 | 1 (0.7) | - | - |
| Respiratory tract infection viral | 3 (3.9) | 3 (2.0) | 9 (1.6) | 22 (2.0) | - | - | - | - |
| Rhinitis | 1 (1.3) | 3 (2.0) | 7 (1.3) | 16 (1.4) | - | - | - | - |
| Sinusitis | 6 (7.8) | 5 (3.4) | 50 (9.0) | 57 (5.1) | - | - | - | - |
| Lower respiratory tract infection | 1 (1.3) | 0 | 9 (1.6) | 17 (1.5) | - | - | - | - |
| Respiratory tract infection | 3 (3.9) | 3 (2.0) | 9 (1.6) | 26 (2.3) | - | - | - | - |
| Respiratory, thoracic, and mediastinal disorders | 12 (15.6) | 21 (14.3) | 93 (16.7) | 172 (15.4) | 1 (1.3) | 2 (1.4) | 15 (2.7) | 26 (2.3) |
| Rhinitis allergic | 5 (6.5) | 8 (5.4) | 26 (4.7) | 31 (2.8) | - | - | - | - |
| *AE*, adverse event; *DPL*, dupilumab; *PBO*, placebo; *SAE*, serious adverse event.  ^†^A neutrophil count of 1500 cells/µL is the lower limit of the normal range, therefore <1500 neutrophils/µL is considered neutropenic. | | | | | | | | |

## **TABLE E2.** Treatment-emergent SAEs in patients with <4000 or ≥ 4000 neutrophils/uL at baseline – safety population

| **Primary system organ class  preferred term, n (%)** | **Neutrophils <4000 cells/µL** | | **Neutrophils ≥4000 cells/µL** | |
| --- | --- | --- | --- | --- |
|  | **Combined PBO  (n = 309)** | **Combined DPL  (n = 612)** | **Combined PBO  (n = 324)** | **Combined DPL**  **(n = 650)** |
| Any class | 11 (3.6) | 23 (3.8) | 13 (4.0) | 17 (2.6) |
| Infections and infestations | 5 (1.6) | 9 (1.5) | 4 (1.2) | 8 (1.2) |
| Abscess | - | - | 0 | 1 (0.2) |
| Bronchitis | 0 | 1 (0.2) | 0 | 1 (0.2) |
| Pneumonia | 1 (0.3) | 3 (0.5) | 1 (0.3) | 1 (0.2) |
| Tick-borne Viral Encephalitis | - | - | 0 | 1 (0.2) |
| Appendicitis | - | - | 0 | 1 (0.2) |
| Atypical Pneumonia | 1 (0.3) | 0 | - | - |
| Capnocytophaga Infection | - | - | 1 (0.3) | 0 |
| Chronic Sinusitis | 0 | 1 (0.2) | - | - |
| Clostridium Difficile Colitis | 0 | 1 (0.2) | - | - |
| Diverticulitis | 1 (0.3) | 0 | - | - |
| Gastroenteritis | 0 | 1 (0.2) | 1 (0.3) | 0 |
| Hepatitis A | 0 | 1 (0.2) | - | - |
| Hepatitis C | - | - | 1 (0.3) | 0 |
| Mastoiditis | - | - | 0 | 1 (0.2) |
| Medical Device Site Infection | 0 | 1 (0.2) | - | - |
| Otitis Media | - | - | 0 | 1 (0.2) |
| Post Procedural Cellulitis | - | - | 0 | 1 (0.2) |
| Pyelonephritis | 0 | 1 (0.2) | - | - |
| Pyelonephritis Chronic | 1 (0.3) | 0 | - | - |
| Upper Respiratory Tract Infection | 1 (0.3) | 0 | - | - |
| Urinary Tract Infection | - | - | 0 | 1 (0.2) |
| Respiratory, thoracic and mediastinal disorders | 7 (2.3) | 18 (2.9) | 9 (2.8) | 10 (1.5) |
| Asthma | 6 (1.9) | 13 (2.1) | 8 (2.5) | 4 (0.6) |
| Pulmonary Embolism | 1 (0.3) | 1 (0.2) | 0 | 1 (0.2) |
| Eosinphilic Pneumonia Chronic | - | - | 0 | 1 (0.2) |
| Dyspnoea | - | - | 0 | 1 (0.2) |
| Haemoptysis | - | - | 1 (1.03) | 0 |
| Interstitial Lung Disease | - | - | 0 | 1 (0.2) |
| Laryngeal Oedema | 0 | 1 (0.2) | - | - |
| Nasal Polyps | - | - | 0 | 1 (0.2) |
| Noninfective Bronchitis | 0 | 1 (0.2) | - | - |
| Pleurisy | - | - | 0 | 1 (0.2) |
| Pneumaothorax Spontaneous | 0 | 1 (0.2) | - | - |
| Respiratory Depression | 0 | 1 (0.2) | - | - |

*DPL*, dupilumab; *PBO*, placebo; *SAE*, serious adverse event.

## **FIGURE E1.** Change in annualized severe exacerbation rates over the treatment period and change from baseline in pre-bronchodilator FEV_1_ at Week 52 by baseline FeNO (ppb) in patients with ≥4000 neutrophils/µL at baseline, and in patients with blood eosinophils ≥150 cells/µL AND ≥4000 neutrophils/µL at baseline.

*CI*, confidence interval; *FeNO*, fractional exhaled nitric oxide; *FEV_1_*, forced expiratory volume in 1 second; *LS*, least squares; *ppb*, parts per billion; *q2w*, every 2 weeks.


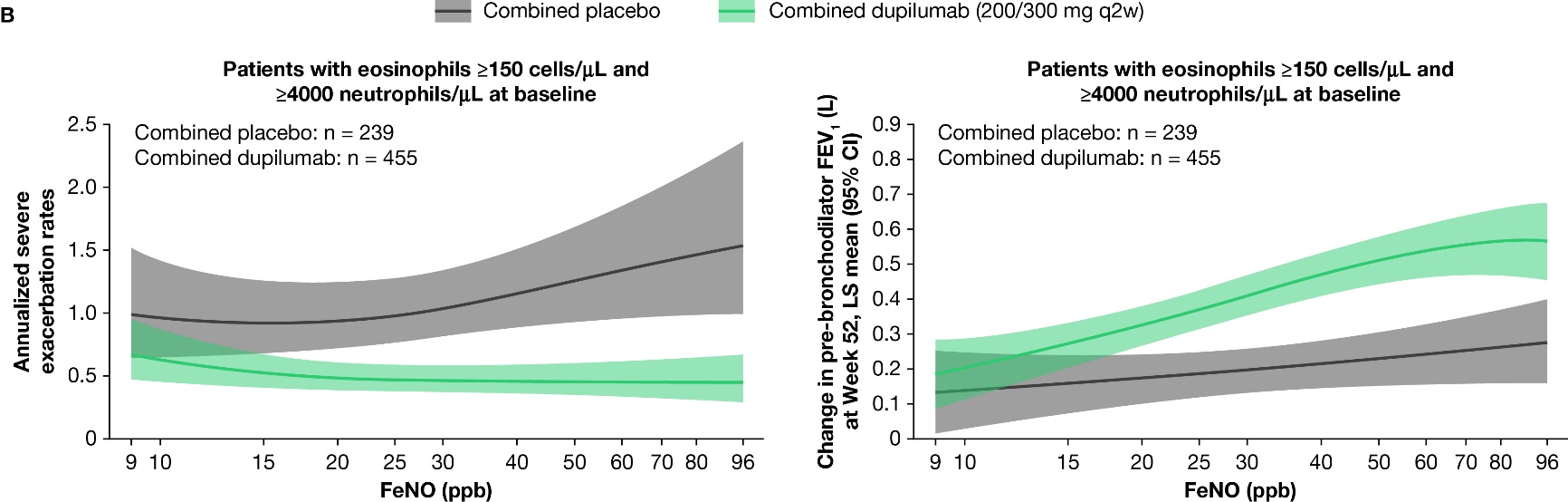

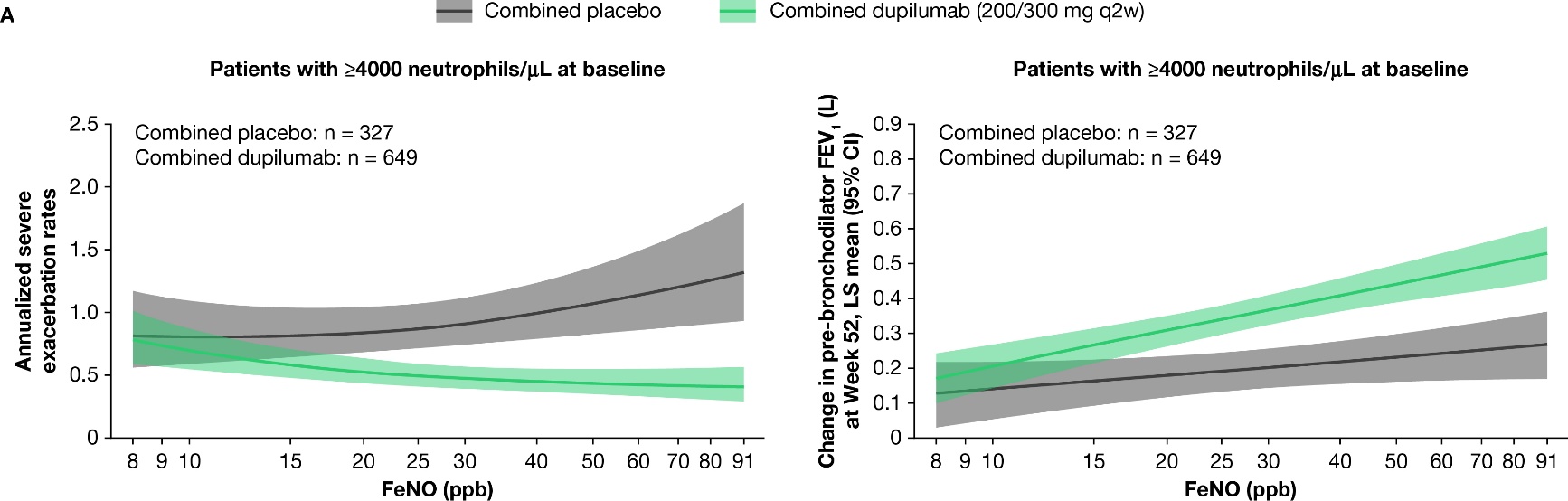


## **FIGURE E2.** Change in annualized severe exacerbation rates over the treatment period and change from baseline in pre-bronchodilator FEV_1_ at Week 52 by baseline FeNO (ppb) in patients with <4000 neutrophils/μL at baseline, and in patients with blood eosinophils ≥150 cells/µL AND <4000 neutrophils/µL at baseline.

*CI*, confidence interval; *FeNO*, fractional exhaled nitric oxide; *FEV_1_*, forced expiratory volume in 1 second; *LS*, least squares; *ppb*, parts per billion; *q2w*, every 2 weeks.


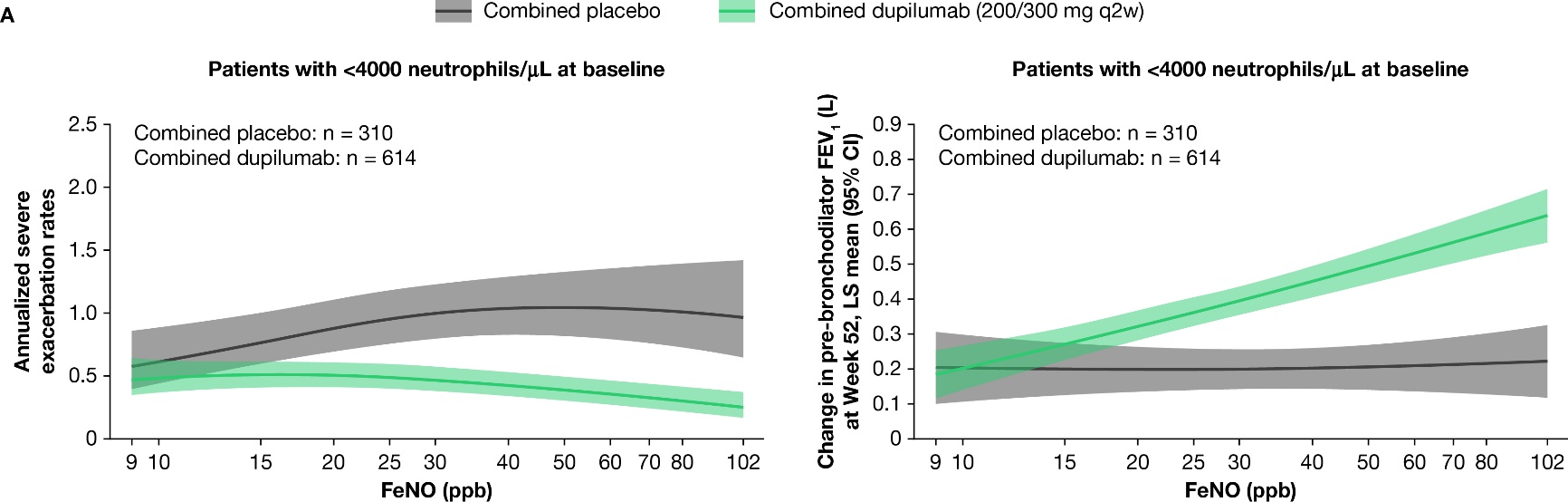

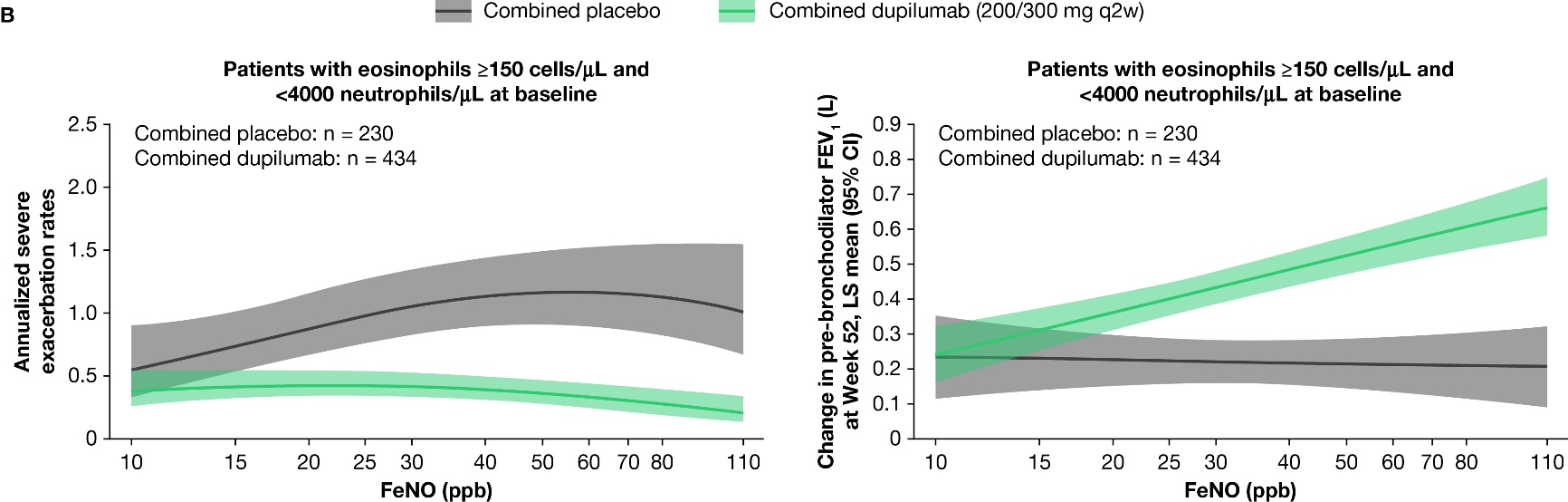

Supplement: Supplementary Materials — Table S1: Treatment-emergent AEs and SAEs in patients with <1,500 neutrophils/µL (neutropenia) or ≥1,500 neutrophils/µL at baseline—safety population. Table S2: Treatment-emergent SAEs in patients with <4,000 or ≥4,000 neutrophils/µL at baseline—safety population. Figure S1: Change in annualized severe exacerbation rates over the treatment period and change from baseline in prebronchodilator FEV1 at Week 52 by baseline FeNO (ppb) in patients with ≥4,000 neutrophils/µL at baseline, and in patients with blood eosinophils ≥ 150 cells/µL AND ≥4,000 neutrophils/µL at baseline. Figure S2: Change in annualized severe exacerbation rates over the treatment period and change from baseline in prebronchodilator FEV1 at Week 52 by baseline FeNO (ppb) in patients with <4,000 neutrophils/μL at baseline, and in patients with blood eosinophils ≥ 150 cells/µL AND <4,000 neutrophils/µL at baseline. [file 9943584.f1.docx]
